# Maintenance of familiarity and social bonding via communal latrine use in a solitary primate (*Lepilemur leucopus*)

**DOI:** 10.1007/s00265-014-1810-z

**Published:** 2014-10-16

**Authors:** Iris Dröscher, Peter M. Kappeler

**Affiliations:** 1 Behavioral Ecology & Sociobiology Unit, German Primate Center, Kellnerweg 4, 37077 Göttingen, Germany; 2 Department of Sociobiology/Anthropology, Johann-Friedrich-Blumenbach Institute of Zoology & Anthropology, University of Göttingen, Kellnerweg 6, 37077 Göttingen, Germany

**Keywords:** Latrines, Olfactory communication, Scent marking, Intra-group communication, Mate defense, Primate

## Abstract

Latrine use (i.e., the repeated use of specific defecation/urination sites) has been described for several mammals, including carnivores, ungulates, and primates. However, the functional significance of latrine use in primates has not been studied systematically yet. We, therefore, followed 14 radio-collared individuals of the pair-living white-footed sportive lemur (*Lepilemur leucopus*) for 1097 hours of continuous focal observations to investigate latrine distribution, seasonality of latrine use, as well as age and sex of users to test various hypotheses related to possible functions of latrine use, including territory demarcation, resource defense, signaling of reproductive state, social bonding, and mate defense. All individuals of a social unit exhibited communal use of latrines located in the core area of their territory, supporting the social boding hypothesis. Latrine use seems to facilitate familiarity and social bonding within social units via olfactory communication in this primate that lives in family units but exhibits low levels of spatial cohesion and direct social interactions. In addition, frequency of latrine visitation was higher during nights of perceived intruder pressure, supporting the mate defense hypothesis. However, animals did not react to experimentally introduced feces from neighboring or strange social units, indicating that urine may be the more important component of latrines than feces in this arboreal species. Based on a survey of latrine use and function in other mammals, we conclude that latrines facilitate communication particularly in nocturnal species with limited habitat visibility and in species where individuals are not permanently cohesive because they constitute predictable areas for information exchange.

**Electronic supplementary material:**

The online version of this article (doi:10.1007/s00265-014-1810-z) contains supplementary material, which is available to authorized users.

## Introduction

Chemical signals can transmit a variety of information in vertebrates, such as species identity (Caspers et al. 2009), sexual identity (Ferkin and Johnston 1995), reproductive state (Ziegler 2013), and individual identity (Linklater et al. 2013). Many chemical signals derive from various excretory products, such as feces, urine, and gland secretions (Eisenberg and Kleiman 1972), and scent marking is defined as the application of these products to features in the environment (Macdonald 1980). The repeated use of specific locations for defecation/urination can result in an accumulation of feces and other excretory products at so-called latrine sites, and this behavior can be considered a special form of scent marking in cases where it serves a communicatory function (Wronski et al. 2013). Latrines have been described for several ungulates (e.g., *Ourebia*: Brashares and Arcese 1999; *Tragelaphus*: Apio et al. 2006; *Mazama*: Black-Decima and Santana 2011; *Gazella*: Wronski et al. 2013), carnivores (e.g., *Suricata*: Jordan et al. 2007; *Vulpes*: Darden et al. 2008; *Meles*: Kilshaw et al. 2009; *Hyaena*: Hulsman et al. 2010), primates (e.g., *Lepilemur*: Charles-Dominique and Hladik 1971; *Cheirogaleus*: Schilling 1980a; *Hapalemur*: Irwin et al. 2004), and a few other mammalian taxa (e.g., *Arvicola*: Woodroffe and Lawton 1990; *Oryctolagus*: Sneddon 1991). Feces are either deposited alone (e.g., *Bassariscus astutus*: Barja and List 2006; *Ourebia ourebi*: Brashares and Arcese 1999) or together with urine and/or secretions of specialized glands at latrine sites (e.g., *Meles meles*: Roper et al. 1986; *Mazama gouazoubira*: Black-Decima and Santana 2011). In several species (e.g., *Vulpes velox*: Darden et al. 2008; *Hyaena* spp.: Gorman and Mills 1984; *Meles meles*: Stewart et al. 2002), urination is the most common mark used in this context, and feces per se may not be the most important information component of a latrine (Darden et al. 2008). Similarly, for arboreal species, one could reasonably expect that any potential communicatory function may be rather related to olfactory signals obtainable from arboreally deposited urine than from terrestrial accumulation of feces, which may rather be a byproduct of localized urine marking.

Among primates, the lemurs of Madagascar (Lemuriformes) represent a radiation whose members rely heavily on chemical signals for their social communication (Mertl 1976; Schilling 1979, 1980b; Perret 1992; Kappeler 1998; Heymann 2006b; Charpentier et al. 2008, 2010; Boulet et al. 2009, 2010; Crawford et al. 2009; Morelli et al. 2013), irrespective of their social organization (Kappeler and van Schaik 2002). The more than 20 species of sportive lemurs (genus *Lepilemur*) are all medium-sized nocturnal folivores. Like many other nocturnal lemurs, they exhibit urine marking (Schilling 1979, 1980b; Epple 1986). In addition, *Lepilemur* males possess anogenital scent glands, while females have no scent glands (Petter et al. 1977; Schilling 1979). Sportive lemurs are strictly arboreal, and patterns of defecation/urination produce terrestrial accumulations of feces (Charles-Dominique and Hladik 1971; Russel 1977; Irwin et al. 2004). Some species live in dispersed pairs, which are characterized by spatial overlap between one adult male and one adult female, but low cohesion between pair partners (Schülke and Kappeler 2003; Zinner et al. 2003; Méndez-Cárdenas and Zimmermann 2009; Hilgartner et al. 2012; Dröscher and Kappeler 2013). Pair partners living in dispersed pairs may never share sleeping sites or allogroom each other, and they may even show signs of active spatial avoidance (Dröscher and Kappeler 2013). In addition, sportive lemurs are highly territorial, as indicated by minimal home range overlap between individuals of neighboring social units (Zinner et al. 2003; Rasoloharijaona et al. 2006; Méndez-Cárdenas and Zimmermann 2009; Dröscher and Kappeler 2013). This combination of traits makes sportive lemurs an interesting taxon to study various potential functions of latrines.

Irwin et al. (2004) reviewed latrine behavior in primates and discussed several hypotheses for the function of latrine use. In particular, they suggested that latrine use in lemurs is mainly linked to the defense of resources, such as specific food patches, mates or sleeping sites. While male sportive lemurs exhibit mate guarding and defend their territories against neighboring males (Hilgartner et al. 2012), they do not defend food resources for their pair mates, and competition for food is low within as well as between social units (Dröscher and Kappeler 2014). However, systematic tests of this potential function of latrines have not been conducted yet.

While latrines may be merely a by-product of a bimodal defecation rhythm that results in the concentration of defecations being deposited under repeatedly used sleeping sites (Julliot 1996; González-Zamora et al. 2012), the use of localized defecation sites can also be explained by several additional, non-exclusive functional hypotheses. Many hypotheses that are commonly formulated for the function of scent marking (e.g., Ralls 1971; Kappeler 1998; Brady and Armitage 1999; Lazaro-Perea et al. 1999; Rostain et al. 2004; Heymann 2006a; Lewis 2006) are also applicable to the function of latrine use, as latrine behavior is a special form of olfactory communication.

In the following, we present hypotheses that are applicable to the social system of our study species (see below) and provide key references for each one of them. First, latrines may be used to demarcate territories, since many mammals are known to use urine, feces or other scent marks to delineate home range boundaries (Mertl-Milhollen 1979; Brashares and Arcese 1999; Stewart et al. 2001; “territory demarcation hypothesis”). Second, latrines may be used to communicate reproductive state, since male mammals seem to be able to detect chemical cues in female urine and/or feces related to reproductive state (Balestrieri et al. 2011; Archunan and Rajagopala 2013; “reproductive signaling hypothesis”). Third, latrines may serve to advertise the willingness to defend important resources such as food (Kruuk 1992; Miller et al. 2003; Remonti et al. 2011) or resting sites (Goszczynski 1990; Branch 1993; Brady and Armitage 1999; “resource defense hypothesis”). Fourth, latrines may function as information exchange centers for individuals that rarely associate or interact directly to facilitate the exchange of olfactory individual-specific information within social units to maintain social bonds (Kingdon 1982; Greene and Drea 2014; “social bonding hypothesis”). Finally, latrines may play a role in mate defense by advertising the commitment of resident males to defend resident females (Roper et al. 1986; Jordan et al. 2007; “mate defense hypothesis”).

By detailing latrine density and distribution, seasonality and behavioral contexts of latrine use as well as age and sex of users, we aimed to test predictions of the above hypotheses. Specifically, (a) if latrines were used to demarcate territories, we expected that they would be located at territorial boundaries or in zones of home range overlap between neighboring social units rather than in core home range areas. (b) If latrines were used to communicate reproductive state, we predicted that frequency of latrine use would increase during the pronounced annual mating season. (c) If latrines were used to contribute to resource defense, we anticipated that latrines would be located in proximity to regular sleeping trees, that feeding effort would be higher within than outside the latrine area, and/or that animals would mark specific food trees by defecation/urination. (d) If latrines were used as information exchange centers for intra-group communication in a species in which individuals of a given social unit visit latrines independently, we expected all individuals of a social unit to visit the same latrines to facilitate information transfer. In addition, we predicted that latrines would be visited exclusively by individuals of a social unit, but not by individuals of neighboring units. (e) If latrines play a role in mate defense, we expected that the frequency of male latrine use would increase with perceived intruder pressure. In addition, we expected that males would place glandular scent marks preferentially in latrines. Finally, (f) since aggression in *L. leucopus* is directed towards roaming individuals rather than neighbors (Dröscher and Kappeler 2013), we expected individuals to react more strongly to experimentally introduced feces of strange individuals than to those of familiar ones (Ydenberg et al. 1988; Müller and Manser 2007).

## Methods

### Study site and animal capture

We studied a population of white-footed sportive lemurs (*Lepilemur leucopus*) at Berenty (S 25.00°, E 46.30°), an approximately 200 km^2^ private ecotourism reserve in southern Madagascar. We observed animals in a spiny forest fragment of about 5 ha (HAH Reserve Forestière parcel 1), which is connected to gallery forest on one side via a transitional forest and a further 40 ha spiny forest fragment on the other side (Norscia and Palagi 2008). To ensure continuing focal observations of single individuals, we equipped animals with radio-tracking transmitters. We used a blowpipe and 1 ml air pressured narcotic syringe projectiles (Telinject, Germany) to anesthetize animals with 0.4 ml Ketanest (100 mg/ml) in the mornings in their daytime sleeping sites. We fitted the animals with radio-collars (TW-3 button-cell tags, Biotrack, UK) while anesthetized. We kept the animals in an animal transport box until they were fully recovered and released them again at their capture site in the evening. We fitted 16 adult (eight males and eight females) and four subadult individuals (three males and one female) with radio-collars. We differentiated adult individuals from subadults by the degree of tooth wear and body mass. We did not radio-collar animals when radio-collars exceeded 4 % of their body mass. We removed all radio-collars after the end of the study. The research followed standard protocols for animal handling, capture, and radio-tracking and was approved by the Commission Tripartite CAFF of the Ministry for Water and Forests (Madagascar).

### Behavioral observations

We collected behavioral and locational data between October 2011 and October 2012 for a total of 1530 hours on 20 radio-collared individuals. For the present study, we only considered focal individuals that were adult and belonged to social units in which both pair mates were radio-collared (*N* = 14 individuals, observation time in sight = 1097 hours). Five out of seven social units consisted of pairs; whereas in the remaining cases, an adult male was associated with two adult females each (social unit 1 and 3). However, these females had exclusive ranges since they were regularly seen within the range of the associated adult male, but never within the range of the other adult female. No behavioral observations could be conducted on these females because they were not equipped with radio-collars. For a detailed description on the identification of the social units within the study population see Dröscher and Kappeler (2013).

We divided the study period into four biologically relevant seasons: birth and offspring care with lactation (early wet season from November to January), offspring care without lactation (late wet season from February to April), mating and early gestation (early dry season from May to July) and late gestation (late dry season from August to October). Each individual was watched for two full nights during each season, once by the first author and once by a Malagasy research assistant, using a TR-4 receiver and a RA-14K antenna (Telonics, USA; Appendix A) to locate animals. However, we included data only for 7 observation nights for male m9 since he joined female f2 only after he displaced the previous resident male. Similarly, we include data only for 4 observation nights for male m10 since he only joined female f1B at the beginning of the mating season.

The trees of the spiny forest have small and exposed canopies (Grubb 2003), permitting nocturnal observation of the subjects clearly and continuously (Hladik and Charles-Dominique 1974). We started continuous focal animal observations (Altmann 1974) when an animal left its sleeping site at dusk until it returned to its daytime sleeping site at dawn. Usually, when the first author watched an adult male, the Malagasy research assistant watched the corresponding adult female during the same night simultaneously and vice versa. An overview of the focal animal observations is given in Appendix A. We tagged spatial locations of animals during continuous focal observations with biodegradable tape while recording the beginning and end of each behavior (i.e., resting, travelling, grooming, feeding, displaying, social interactions). We determined the exact position of the tagged trees with reference to a 10 × 10m study grid system. In addition, we recorded all occurrences of defecation, urination, scent marking (i.e., rubbing of the anogenital region on a substrate) and olfactory inspection (i.e., sniffing and licking of substrate) of the focal animals along with their spatial location. We distinguished between single-use and multiple-use defecation sites by investigating the degree of ground coverage by feces (a few scattered droppings that could have been produced by a single defecation event vs. concentrated accumulation of feces indicative of multiple use). In addition, ID recorded the same data every time she could observe an un-collared animal defecating/urinating. Each morning after a full-night follow, we located the sleeping trees of all radio-collared animals.

### Experimental translocation of feces

To establish whether animals discriminate between feces of their own, neighboring and strange social units, we conducted latrine translocation experiments in June 2013 with males and females of 5 social units. We gathered feces from latrines from known neighboring social units (i.e., “neighbor treatment”) and from latrines we located in a neighboring forest parcel, to ensure that the feces originated from social units that were not familiar to the focal animals (i.e., “stranger treatment”). Similarly, we gathered feces from latrines of the focal social unit (i.e., “control treatment”). For the experiments, we spread the gathered feces on plastic sheets of approximately 1 m^2^ (i.e., “experimental latrine”). We handled the feces using disposable plastic gloves. To ensure that the focal animals would encounter the experimental latrines, we determined through preliminary observations which latrine tree each of the focal animals would visit first after leaving the day-time resting tree. For the experiments, we introduced the feces in proximity to the identified latrine tree before sunset. For each experiment, we used an approximately equal amount of feces. We started to record behavioral responses (i.e., loud calling, displaying, glandular scent marking, and sniffing) from the moment the focal individual entered the experimental latrine tree and continued behavioral observations for 30 min. In addition, we recorded the amount of time the animal spent in the latrine tree. We randomized the order in which we presented the three experimental treatments to the focal individuals. We only conducted one experimental treatment on one social unit during a single night. We removed the plastic sheets with the experimental feces immediately after each experiment.

### Data analyses

To determine whether animals discriminate between feces of their own, neighboring and strange social units, we used Friedman’s ANOVA to test for differences between experimental treatments. We used rates of loud calling, sniffing, displaying, and glandular scent marking as measures of response intensity in males, but only rates of loud calling and sniffing in females. A new bout started when an individual interrupted the behavior for more than 5 s. In addition, we used the amount of time the animals spent in the experimental latrine tree as a response variable in both sexes. We based all calculations on the time the animals were in sight.

To establish the number and to investigate the distribution of latrines within the territories of the 7 social units, we calculated the size of individual annual home ranges with the Animal Movement extension of ArcView and plotted all recorded defecation/urination events. Since kernel densities do not require serial independence of observations, we did not correct for spatial autocorrelation (De Solla et al. 1999). However, we based our home range estimates on a constant time interval (i.e., 5 min) that is biologically meaningful, since it allows individuals to traverse their home range at maximum speed (Rooney et al. 1998). We calculated home range size from 95 % fixed kernel home range utilization distributions (Worton 1989) using ad hoc smoothing (Silverman 1986). To establish whether defecation/urination occurred anywhere in an animal’s home range (i.e., random distribution of events) or were restricted to certain areas (i.e., clumped distribution of events), we used the nearest neighbor analysis as implemented in the Animal Movement extension for ArcView (Hooge and Eichenlaub 1997). While *R* values of 1 indicate a random distribution, values of <1 and >1 indicate a tendency towards a clumped or a uniform distribution, respectively. Before running the analyses, we applied a small amount of random noise to the spatial location points of observed defecation/urination events to break ties between repeated observations at the same localities using the function “jitter” of the R software (R Core Team 2012).

After ascertaining the spatial distribution of defecation/urination events via nearest neighbor analysis as being clumped, we established the number of latrines per territory by visual inspection of the spatial features in ArcView. Specifically, we considered a latrine as a cluster of defecation/urination events that were at a distance of up to 6 m of each other. We choose 6 m as a distance criterion because this was the minimum distance at which a cluster of defecation/urination events would not disintegrate in a larger number of smaller, non-continuous latrines in close proximity to each other. When testing the various functional hypotheses of latrine use, we only considered defecation/urination events that were clearly associated with latrine visitations by removing all random defecation/urination events (i.e., single-use defecation sites that were not in proximity to a latrine; *N* = 32 or 5 % of all defecation/urination events recorded).

To test the territory demarcation hypothesis, we established the number of defecation/urination events within the core vs. the boundary area as well as in the zones of home range overlap. We delineated core areas using a time maximizing function derived from kernel analyses (Vander Wal and Rodgers 2012).

To test the resource defense hypothesis with regard to defense of food, we investigated whether animals spent less time feeding within than outside the latrine area. We defined food patches as single feeding trees in which animals were observed feeding. Each food patch that was located within 6 m of a latrine tree was assigned as being part of the general latrine area. We calculated the relative proportion of feeding time within and outside the latrine area for each focal individual. In addition, we calculated the relative proportion of the number of food patches located within and without the latrine area. We calculated an index of feeding effort that allows accounting for the fact that the latrine area is smaller than the remaining home range area, and hence, innately can only contain a smaller number of potential food patches. We divided the proportion of foraging time within the latrine area by the relative proportion of the number of food patches located within the latrine area to calculate an index of feeding effort inside the latrine area. Likewise, we divided the proportion of foraging time outside the latrine area by the relative proportion of the number of food patches located outside the latrine area to calculate an index of feeding effort outside the latrine area. We compared feeding effort within and outside the latrine area using Wilcoxon signed-ranks test for each focal individual.

To test the resource defense hypothesis with regard to defense of sleeping sites, we investigated spatial dependence between defecation/urination sites and regular sleeping sites (i.e., sleeping trees that were used more than once by the focal animals). We conducted the analyses using the R package “spatsat” (Baddeley and Turner 2005). We defined the union home range of all study individuals as the sampling window. We used the L-cross function to describe the dependence in bivariate point patterns using the independence approach (Dixon 2002). We used the inhomogeneous L-cross function to adjust for spatially varying intensity. For formal hypothesis testing, we computed simulation envelopes by pointwise Monte Carlo test. We used 99 simulations of CSR (complete spatial randomness) to compute envelopes. The theory of the Monte Carlo test requires the distance (*r*) to be fixed in advance for hypothesis testing (Baddeley and Turner 2005). We used a value of 6 m as a critical distance. Spatial dependence between points of two types occurs when events of each type are either closer (clustering) or farther away (inhibition) than expected under the assumption that the two processes are independent. Likewise, to test the mate defense hypothesis we investigated spatial dependence between defecation/urination sites and male glandular scent marking sites.

To test the reproductive signaling hypothesis, we used linear mixed models (LMM) to estimate the effect of season on latrine use frequency (model 1). Since season may have a different effect on latrine use frequency in the two sexes, we included season, sex, and their interaction in the model. We included individual identity nested within social unit as a random effect to control for pseudo-replication. In addition, to test the mate defense hypothesis, we used LMM to estimate the effect of intruder pressure on latrine use frequency in males (model 2). We considered observation nights in which focal males engaged in display behavior (i.e., branch bashing displays accompanied by loud calling) and/or placed glandular scent marks as nights with perceived intruder pressure. For each full-night observation, we calculated the frequency of latrine use by dividing the number of latrine visits by the amount of time the focal animal was in sight. We included individual identity as a random effect to control for repeated observations. We controlled for the effect of the number of latrines within an individual’s home range as well as for the effect of the type of social organization the individual lived in (i.e., pairs vs. one-male, two-female units). We transformed response variables using the function “boxcox” of the package “MASS” (Venables and Ripley 2002) and *z*-transformed the covariate (i.e., number of latrines; Schielzeth 2010).

We checked the distribution of the model residuals, plotted residuals against predicted values, conducted the Levène’s test and correlated absolute residuals with fitted values to check model validity. We visually inspected qq-plots and plots of residuals vs. fitted values. None of the diagnostics indicated deviations from the assumptions of normality and homogeneity of residuals (Quinn and Keough 2002; Field et al. 2012). We calculated Variance Inflation Factors (VIFs) using the R function “vif” of the package “car” (Fox and Weisberg 2011) running a standard linear model with the random effect excluded from the predictors. VIFs indicated collinearity not to be an issue (largest VIF for model 1 = 2.03 and for model 2 = 1.35, respectively; Field et al. 2012). For influence diagnostics (Cook’s distance, dfbetas), we used the R package “influence.ME” for mixed effect models (Nieuwenhuis et al. 2012). The largest Cook’s distance was only 0.14 for model 1. However, Cook’s distances indicated some problems with model stability for model 2 (largest Cook’s distance = 1.55). Similarly, unstandardized DFBeta values reached 1.15 for model 2, whereas values did not indicate any problems for model 1 (largest DFBeta = 0.68; Quinn and Keough 2002; Field et al. 2012). Running the second model without the influential case (male 4) did not lead to a different overall result, and hence, we report the results obtained for the complete dataset. To test whether season or intruder pressure, respectively, had an overall effect on latrine use frequency we compared the full model to a model in which only these predictors were removed (i.e., season and its interaction with sex or perceived intruder pressure, respectively), using a likelihood ratio test. We fitted the models in *R* using the function “lmer” in the package “lme4” (Bates et al. 2012) using Maximum Likelihood rather than Restricted Maximum Likelihood to achieve more reliable *P* values (Bolker et al. 2008). We derived *P* values for the individual effects based on Satterthwaite approximation for denominator degrees of freedom by using the function “summary” of the R package “lmerTest” (Kuznetsova et al. 2014). We considered *P* ≤ 0.05 as statistically significant.

## Results

### General latrine behavior

Animals remained on average 5.8 ± 9.4 min (mean ± SD; *N* = 678) in trees in which they defecated/urinated. Similarly, they spent in total only 6 % of the total observation time they were in sight in trees in which they defecated/urinated. They lifted their tail to defecate and urinate while clinging to tree trunks. While the feces dropped to the ground, the urine dripped down the main trunk of the tree and left visible stains even once the urine was dried. While *Lepilemur* feces were not very odorous, at least to the human nose, urine was characterized by a distinct species-specific odor. We could observe the focal animals on two occasions to lick and on 26 occasions to sniff the bark of a tree. On 15 of these occasions this behavior occurred in the general latrine area and on six occasions in an identified latrine tree. Outside the observation period, we could observe a male to sniff a wet urine stain that was deposited 8 min earlier by a female in the latrine. In addition, we could observe the animals on four occasions to lower themselves to less than 1 m above the ground in a latrine tree to inspect the ground.

### Experimental translocation of feces

The time spent in the experimental latrine ranged between 11 % and 80 % (mean ± SD = 29 ± 23) of the observation time in females and between 11 and 39 % (20 ± 7) in males. Rates of loud calling ranged between 0 and 2 bouts/h in females (0.14 ± 0.55) and males (0.27 ± 70). While we could not observe females to engage in sniffing, rates of sniffing ranged between 0 and 8 bouts/h in males (1.21 ± 2.49). We could not observe males to engage in display behavior during the experiment, but rates of scent marking ranged between 0 and 2 bouts/h (0.54 ± 0.92). Response intensity did not differ significantly among the three experimental treatments in either males or females. More precisely, the amount of time spent in the latrine tree (females, *χ* ^2^ = 1.3, *df* = 2, *P* = 0.522; males, *χ* ^2^ = 5.7, *df* = 2, *P* = 0.058), rates of loud calling (females, *χ* ^2^ = 0.3, *df* = 2, *P* = 0.861; males, *χ* ^2^ = 0.3, *df* = 2, *P* = 0.861), sniffing (females, *χ* ^2^ = 0.0, *df* = 2, *P* = 1; males, *χ* ^2^ = 1.2, *df* = 2, *P* = 0.549), displaying (males, *χ* ^2^ = 0.0, *df* = 2, *P* = 1), and scent marking (males, *χ* ^2^ = 1.2, *df* = 2, *P* = 0.549) did not differ significantly among treatments.

### Spatial distribution of defecation/urination events

**Fig. 1:**
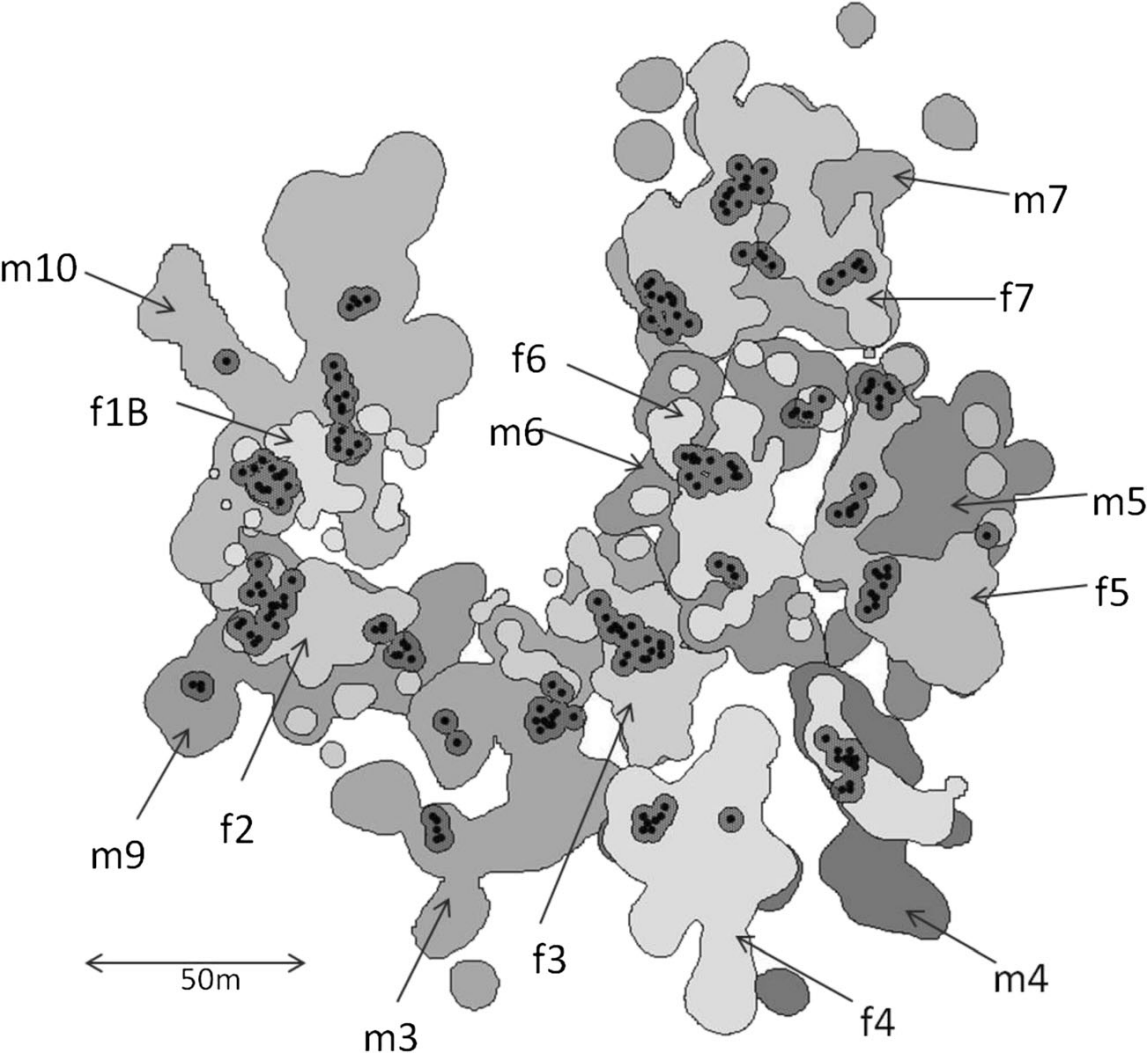
Ninety-five percent kernel annual home ranges for individual adult males (*m*) and females (*f*) of *Lepilemur leucopus* at Berenty between October 2011 and October 2012 as well as the spatial arrangement of the latrines within the home ranges. *Dots* represent individual latrines trees, whereas the *shaded areas* represent a contagious buffer of 3 m around individual latrine trees to distinguish discrete latrines. Home ranges of pair partners overlap (Sex, *m* = male, *f* = female)

Union home range size (95 % Kernel estimates) for the seven social units ranged between 0.28 and 0.47 ha (mean ± SD = 0.38 ± 0.07 ha, *N* = 7). Nearest neighbor analyses of the locations of defecation/urination events computed *R* values ranging between 0.15 and 0.48 for the union home ranges. Within all seven union home ranges the spatial distribution of the defecation/urination events differed significantly from a random spatial distribution (*P* < 0.001, *N* = 7), with a tendency towards clumping as opposed to towards an even distribution (Table 1). We identified 3 to 4 latrines in each union home range (Fig. 1).

**Table 1:** Spatial distribution of observed defecation/urination events within the union home ranges of seven social units of *Lepilemur leucopus* based on nearest neighbor analysis

Social unit	# defecation events	*Z* value	*R* value	*P* value
1	100	−13.59	0.22	<0.001
2	135	−16.73	0.17	<0.001
3	112	−12.75	0.23	<0.001
4	72	−8.09	0.48	<0.001
5	86	−11.34	0.35	<0.001
6	115	−17.20	0.15	<0.001
7	90	−13.91	0.22	<0.001

### Territory demarcation hypothesis

We recorded a total of 678 defecation/urination events. Using the time maximization function, core areas of individual ranges were delineated by 65 % isopleths. Union core areas (65 % Kernel estimates) represented 26 ± 6 % (range = 20–37 %, *N* = 7) of the union home ranges (95 % Kernel estimates) of the social units. However, the majority of defecation/urination events (mean ± SD = 82 ± 7 %; range = 72–94 %, *N* = 7) were located within the small union core areas of the social units, so that the density of defecation/urination events was significantly higher in the core area (mean ± SD = 875 ± 391 events/ha) compared to the remaining home range area (72 ± 54 events/ha; Wilcoxon signed-rank test, *V* = 28, *P* = 0.016, *N* = 7). The overlap zones comprised only 1.35 % of the union of all individual home ranges. None of the defecation/urination events were located within overlap zones of neighboring territories.

### Resource defense hypothesis

The relative proportion of foraging time within the latrine area ranged between 22 % and 43 % (mean ± SD = 31 ± 7 %, *N* = 14). The relative proportion of the number of patches located within the latrine area ranged between 23 % and 46 % (34 ± 7 %). The index of feeding effort inside the latrine area ranged between 0.7 and 1.1 (0.9 ± 0.1) and between 0.8 and 1.1 (0.9 ± 0.1) for the feeding effort outside the latrine area. Feeding effort within the latrine area did not differ significantly from the feeding effort outside the latrine area (Wilcoxon signed-ranks test, *V* = 56, *N* = 14, *P* = 0.851). The animals spent only between 2 % and 14 % (mean ± SD = 7 ± 4 %, *N* = 14) of the total feeding time eating in identified latrine trees. While we could record a total number of 1,584 food patches throughout the study, animals were only seen to defecate/urinate in 79 of them. In addition, animals were observed to forage in only 41 % ± 11 % (range = 24 % - 55 %, *N* = 14) of the identified latrines trees.

**Fig. 2:**
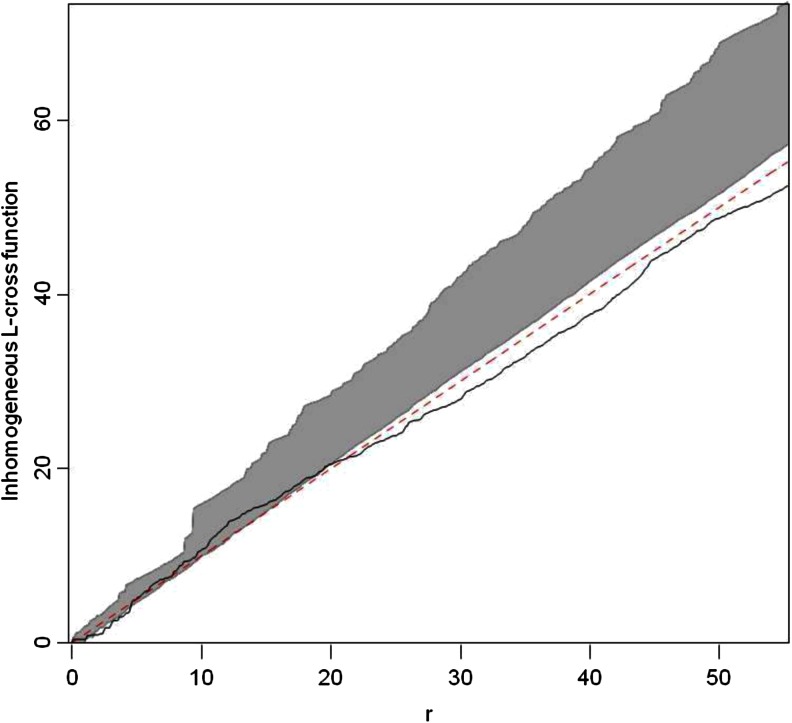
Estimated inhomogeneous L-cross function and envelopes for the bivariate point pattern consisting of defecation/urination sites and sleeping trees. The *solid line* indicates the empirical L-cross function, the *dotted line* indicates the theoretical value for complete spatial randomness (CSR), and the *gray band* indicates the envelope from 99 simulations and *r* is the distance argument

The number of repeatedly used sleeping trees ranged between 5 and 10 (mean ± SD = 7 ± 2) for the 7 social units. None of the latrine trees served as a sleeping tree. The computed empirical homogenous L-cross function fell within the simulation envelop at the critical distance of 6 m, indicating spatial independence between defecation/urination and sleeping sites (Fig. 2).

### Social bonding hypothesis

Regarding the social units consisting of one adult male and two adult females (unit 1 and 3), all latrines located within the common range of the focal male and focal female were shared by both adult individuals. All latrines within the home ranges of social units consisting of one male and one female were shared by both pair partners, with the exception of social unit 2 where only 2 of 3 latrines were shared. We only once saw a focal individual (m6) to visit a neighbor’s latrine (unit 7). In addition, we recorded 47 defecation/urination events by un-collared individuals. 46 of these defecation/urination events were associated with an identified latrine. In 41 of these cases, it was the offspring, which ranged within the parental territory. In 6 cases, it was the second adult un-collared female of unit 1 and 3, respectively. In total, we could observe co-use by un-collared individuals in 18 out of 25 identified latrines.

### Reproductive signaling hypothesis

Latrine use frequency (number of latrine visitations/h) equaled 0.58 ± 0.25 (mean ± SD; *N* = 25) during the early wet, 0.48 ± 0.21 (*N* = 26) during the late wet, 0.48 ± 0.19 (*N* = 28) during the early dry and 0.55 ± 0.19 (*N* = 28) during the late dry season. The result of the LMM to estimate the effect of season on latrine use frequency (model 1) indicated that the full model containing the effects of season and its interaction with sex was not significantly better in explaining the data than the null model (likelihood ratio test, *χ* ^2^ = 8.639, *df* = 7, *P* = 0.279).

### Mate defense hypothesis

**Fig. 3:**
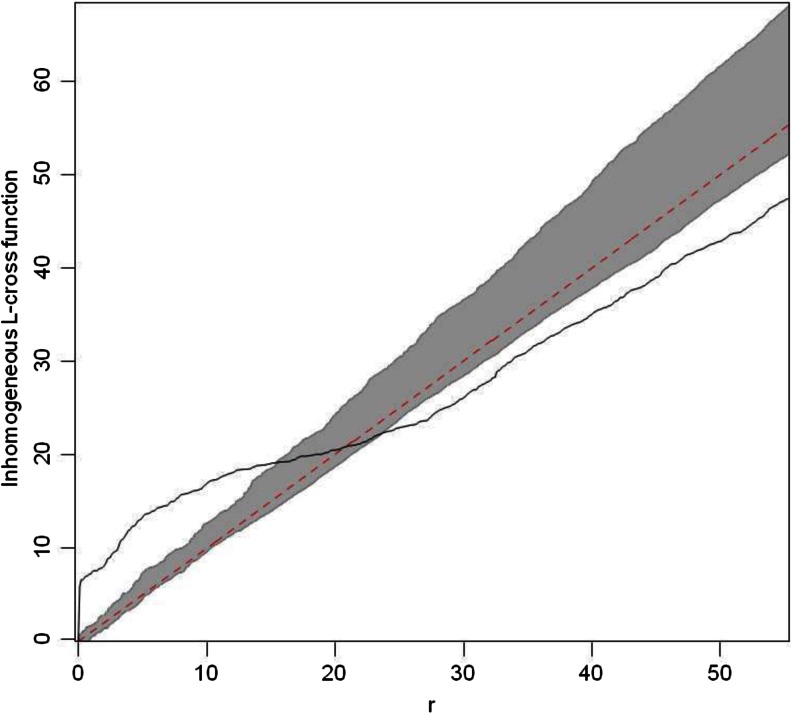
Estimated inhomogeneous L-cross function and envelopes for the bivariate point pattern consisting of defecation/urination and scent marking sites. The *solid line* indicates the empirical L-cross function, the *dotted line* indicates the theoretical value for complete spatial randomness (CSR), the *gray band* indicates the envelope from 99 simulations, and *r* is the distance argument

During 25 observations nights, we observed focal males to place anogenital scent marks and during 21 nights they engaged in branch bashing and vocal displays. One or both of these behaviors were recorded during 37 out of 51 observation nights on adult males. The result of the LMM to estimate the effect of perceived intruder pressure (as indicated by display and scent marking behavior) on latrine use frequency in males (model 2) showed that the full model was significantly better in explaining the data than the null model (likelihood ratio test, *χ* ^2^ = 6.3327, *df* = 1, *P* = 0.012). Latrine use frequency was significantly increased in males during nights of perceived intruder pressure (mean frequency of latrine visitation ± SD: nights with intruder pressure = 0.60 ± 0.27 latrine visitations/h, nights without intruder pressure = 0.46 ± 0.18; P = 0.011; Table 2). In total, we recorded 50 scent marking events by the 7 focal males. 32 of these scent marks were placed in an identified latrine tree. At the critical distance of 6 m, the computed empirical inhomogeneous L-cross function fell above the simulation envelop, indicating spatial dependence (attraction) between latrines and scent marking locations (Fig. 3).

**Table 2:** Effects of perceived intruder pressure, number of latrines, and social organization on latrine use frequency in male *Lepilemur leucopus* (LMM)

Fixed Factor	β	SE	*df*	*t*	*P*
Intercept	1.019	0.002	9.55	473.012	NA
Intruder pressure perceived (yes)	−0.005	0.002	44.44	−2.658	0.011
Number of latrines	0.003	0.002	6.69	1.474	0.186
Social organization (1 ♂ and 2 ♀)	−0.007	0.004	7.49	−1.672	0.136

## Discussion

Our study revealed that defecation/urination events were highly clustered in space, resulting in 3–4 latrines with terrestrial accumulations of feces in each territory. The study animals spent only a notably short time in trees they visited for defecation/urination, and therefore, the formation of latrines is not a mere by-product of animals remaining for a considerable time in a few preferred resting trees (Charles-Dominique and Hladik 1971; Schilling 1979). The number and locations of latrines were stable throughout the study period. We tested whether terrestrial accumulations of feces in an arboreal species can be considered to have an olfactory signaling function. We found no support for this notion and conclude that urine, which is more accessible to the animals for olfactory investigation, is the more important latrine component in this species. Additionally, we found empirical support for the hypotheses that latrines function in social bonding and mate defense, but a potential function in territory demarcation, resource defense, and signaling of reproductive state could not be shown. Below, we discuss these findings in relation to the social system of *L. leucopus* and in light of available data for other latrine-using mammals.

### Experimental translocation of feces

Most species that exhibit latrine use are terrestrial, and feces are, therefore, assumed to be salient sources of olfactory signals. However, *L. leucopus* did not react differently to experimentally introduced feces from neighboring or strange social units, compared to feces from familiar animals. In contrast, river otters (*Lontra canadensis*) investigate foreign scat more than local one when added to latrines (Oldham and Black 2009). Brown brocket deer (*Mazama gouazoubira*) investigate introduced dung from unknown individuals of the same sex significantly more than their own dung, and males counter-mark introduced dung with a greater frequency than females (Black-Decima and Santana 2011). Badgers (*Meles meles*) respond more intensely towards foreign feces, and the response is greatest during the breeding season (Palphramand and White 2007). Among primates, only *Cheirogaleus* spp*.* produce arboreal latrines by smearing feces on branches during repeated walking defecation, resulting in a fecal accumulation adhering to the branch (Petter 1962). In arboreal species, such as *L. leucopus*, terrestrial latrines may serve as an optical signal (Irwin et al. 2004). Moreover, urination above ground facilitates dispersal of the odor by wind and increases the evaporating surface as the urine drips downward (Sillero-Zubiri and Macdonald 1998). Because urine marking is an ancestral behavior in strepsirrhine primates (Delbarco-Trillo et al. 2001), more experimental studies of urine communication in solitary and nocturnal species are called for.

### Social bonding

Scent marks may function as self-advertisement and simply signal an individual’s presence and identity to mates, family members, neighbors, and/or intruders (Eisenberg and Kleiman 1972; Peters and Mech 1975; Wolff et al. 2002), and latrines may serve as information exchange centers of individual-specific information (Darden et al. 2008; Black-Decima and Santana 2011). Latrines are maintained by all individuals of a social unit in *L. leucopus*. In contrast, in European badgers (*Meles meles*), a species in which latrines function mainly in territorial defense and demarcation, sexually immature juveniles rarely defecate/urinate at latrines (Brown et al. 2009). Latrines have been suggested to help maintaining social bonds in some ungulates such as steenbok (*Raphicerus campestris*), oribi (*Ourebia ourebi*), and dikdik (*Madoqua kirkii*; Kingdon 1982; Apio et al. 2006). Behaviors that facilitate familiarity, and hence, intra-group recognition may be especially important in solitary foragers with minimal direct social contact between individuals (Dröscher and Kappeler 2013). In contrast, mated pairs of swift foxes (*Vulpes velox*) exhibit high levels of den sharing that allows the exchange of information within the pair and to maintain the pair bond. Thus, latrines are not considered important for intra-pair communication and maintenance of social cohesion in *V. velox* (Darden et al. 2008). Latrine locations within the core areas of *L. leucopus* also support the idea that they function in social bonding since this form of placement should be particularly suited for information exchange between group members (Wronski et al. 2013).

In Coquerel’s sifakas (*Propithecus coquereli*), the quality of the pair bond of breeding pairs is reflected in their olfactory signals by chemical convergence, possibly due to similar volatile production by shared microbial communities obtained through the exchange of odorant-producing microbes for example via overmarking (Greene and Drea 2014). Similarly, anal gland secretions that coat or saturate badger feces seem to have a group-specific chemical composition (Davies et al. 1988). Analogously, convergence in vocal signals facilitates group and pair cohesion in some primate and avian species (Geissmann and Orgeldinger 2000; Tyack 2008; Sewall 2009; Candiotti et al. 2012). Sportive lemurs not only exchange chemical but also acoustic signals. While pairs of the Milne Edwards’ sportive lemur (*L. edwardsi*) coordinate loud calls in duets, perhaps to strengthen pair bonds (Méndez-Cárdenas and Zimmermann 2009), neither red-tailed sportive lemurs (*L. ruficaudatus*; Fichtel and Hilgartner 2013) nor *L. leucopus* exchange vocalizations in coordinated duets. In addition, males and females of *L. leucopus* produce sex-specific loud calls and thus are not available for vocal convergence. It, therefore, remains to be determined what exactly social bonding entails in different species and which aspects of it can be communicated in different modalities.

### Mate defense

Latrines may play a role in mate defense by advertising the commitment of resident males to defend co-resident females (Roper et al. 1986; Jordan et al. 2007). We found that male latrine use frequency increased during nights of perceived intruder pressure. Likewise, latrine use frequency increases in meerkats (*Suricata suricatta*) when prospecting males are present (Jordan et al. 2007). In European badgers (*Meles meles*), males visit boundary latrines more often than females (Roper et al. 1993; Stewart et al. 2001), presumably to signal their commitment to guarding females of their own social group (Roper et al. 1986). Similarly, male brown brocket deer defecate/urinate more often after detecting dung from unknown individuals near one of their latrines. By re-marking their latrine, residents are thought to affirm their dominant or resident status (Black-Decima and Santana 2011).

We do not have systematic data on the behavior of intruders. However, outside the focal observation period, we could observe a resident and a roaming male to repeatedly visit the same latrine tree to defecate, urinate and place glandular scent marks. Male scent marking is linked to intra-sexual competition in several species (e.g., *Microtu* sp.: Jannett 1986; *Myocastor coypus*: Gosling and Wright 1994; *Lemur catta*: Kappeler 1998), and by strategically placing anogenital scent marks in latrines, which are composite olfactory signals of all members of a group, males of *L. leucopus* may also signal their competitive ability and willingness to defend their social unit to intruders (Rich and Hurst 1998).

### Signaling of reproductive state

Males are often able to detect chemical cues in female urine and/or feces related to reproductive state (Rasmussen et al. 1982; Ghosal et al. 2012; Archunan and Rajagopala 2013). Contrary to our predictions, frequency of latrine use in *L. leucopus* did not increase during the mating season. In contrast, genets (*Genetta genetta*) exhibit increased scat deposition at latrine sites during the mating period (Barrientos 2006). Similarly, latrine visitation peaks during the mating season in *M. meles* (Pigozzi 1989; Roper et al. 1993). While females may scent mark to advertise their reproductive state to attract males (Converse et al. 1995; Heymann 1998; Kappeler 1998), males may mask female scent to hide their oestrous condition from competing males or to advertise their presence to other males (Trumler 1958; Klingel 1974; Rich and Hurst 1998; Lewis 2005; Jordan et al. 2007). Although we cannot exclude the possibility that reproductive status may be communicated at latrine sites in *L. leucopus*, the function of latrine use does not appear to be specifically related to male attraction or to over-marking signals of estrous females, since neither females nor males increased latrine use frequency during the mating season. However, estrus in sportive lemurs is seasonal and short (Randrianambinina et al. 2007; Hilgartner et al. 2008) and any effect may have been concealed by our method of data collection, because we did not follow pairs when females were apparently in estrus.

### Territory demarcation

Urine and feces are common, readily available materials and many mammals use them to demarcate their territories or home ranges (e.g., *Meles meles*; Pigozzi 1989; *Panthera tigris*: Smith et al. 1989; *Ourebia ourebi*: Brashares and Arcese 1999). We found that the majority of defecation/urination events were localized within the core areas of the territories, even though *L. leucopus* is highly territorial (Dröscher and Kappeler 2013). However, where latrines cannot be economically maintained because territory borders are too long, they should be placed in the centre of the territory (Jordan et al. 2007). For example, brown hyenas (*Hyaena brunnea*) exhibit boundary marking when they live in small territories but display center marking if they inhabit large territories (Mills and Gorman 1987). Since territory size in *L. leucopus* is only 0.3 ha and individuals can easily traverse their territories in no more than 5 min, it is unlikely that territory size in this species would preclude a border marking strategy. In *M. meles*, latrine use is primarily concentrated along territory boundaries and these are shared by members of the same and neighboring groups (Kilshaw et al. 2009) and are visited mainly by males (Roper et al. 1993). Besides boundary latrines, badgers also use hinterland latrines, which are visited by both sexes (Roper et al. 1993). In *L. leucopus*, all latrines were visited by both pair partners. Furthermore, we could observe only once a focal animal to visit a neighboring latrine, indicating that latrines in *L. leucopus* are not used for inter-group information transfer to monitor occupancy of surrounding territories (Jordan et al. 2007). Instead of latrines, sportive lemurs seem to use vocalizations to signal occupancy and to regulate spacing within and between social units (Rasoloharijaona et al. 2006; Fichtel and Hilgartner 2013).

### Resource defense

Resources such as resting sites (Goszczynski 1990; Branch 1993; Brady and Armitage 1999) and food trees may be marked to identify ownership and to deter conspecifics (Kruuk 1992; Miller et al. 2003). Contrary to our prediction, spatial locations of latrine trees and sleeping trees were spatially independent from each other, notwithstanding the fact that sportive lemurs only use a few selected sleeping sites and appropriate sleeping sites are limited, potentially leading to competition within or between social units (Rasoloharijaona et al. 2003, 2008). Establishing ownership of sleeping sites, therefore, may be beneficial to individuals by ensuring protection from predators or adverse climatic conditions (Franklin et al. 2007). For example, weasel sportive lemurs (*L. mustelinus*) gouge trees after leaving sleeping sites and before moving around, suggesting that they use non-nutritive tree gouging to display ownership of sleeping sites (Rasoloharijaona et al. 2010). Tree gouging behavior is absent in *L. leucopus*, and if latrines were to function instead for sleeping site defense, one would expect latrine trees to be in proximity to sleeping trees. Conversely, scent marks can potentially be exploited by predators to localize prey (Cushing 1984; Viitala et al. 1995), and an intentional placement of latrine trees in proximity to sleeping trees would seem to be disadvantageous in terms of predator attraction. In addition, animals may mark food trees as a means of asserting ownership of food resources.

Communal use of latrines in *L. leucopus* rejects the idea that they are used to signal resource use among members of a social unit. In contrast, otters (*Lutra lutra*) deposit spraints (i.e., token feces) to signal the use of feeding areas exploited by each individual (Kruuk 1992). Alternatively, members of a social unit of *L. leucopus* may use latrines to signal to other social units their willingness to defend their food resources. However, *L. leucopus* did not preferentially defecate/urinate in food trees since animals were observed to defecate/urinate in only 5 % of all identified food patches and to feed in less than 50 % of the identified latrine trees. In addition, the fact that individual feeding effort was equally distributed within and outside the latrine area indicates that latrines are not used to mark important feeding areas. These results are in line with the observation that *L. leucopus* exhibits low dietary selectivity, relies on the most common food species, and rarely engages in conflict over food neither within nor between social units (Dröscher and Kappeler 2014).

## Conclusions

Latrines are found in solitary, pair-, and group-living mammals (Table 3). Latrine use appears to be common among species that are nocturnal, exhibit a dispersed social system, and are territorial. Since many species do not just defecate, but often also urinate and deposit glandular secrets at latrine sites, these signals may function to convey more than one message. Especially in arboreal species with terrestrial accumulations of feces, urine may be of greater importance for chemical signaling than feces. Despite comparative data being sparse, a general pattern emerges that latrines are used in intra-specific olfactory communication in many cases. Although not restricted to nocturnal species, latrine use may facilitate communication in species with limited habitat visibility. Furthermore, latrines can be considered to be economical in species with low inter-individual cohesion, since individuals can benefit from predictable areas for information exchange. Notwithstanding the fact of being more common among territorial species, latrine use does not appear to necessarily function in territory demarcation. Clearly, more experimental studies are required to investigate the relative importance and functions of different modes of olfactory signaling at latrine sites.

**Table 3:** Overview of mammalian latrine users and species-specific attributes such as habitat use (*T* = terrestrial, *A* = arboreal, *AQ* = aquatic), period of activity (*D* = diurnal, *N* = nocturnal, *C* = crepuscular), social organization (*S* = solitary, *P* = pair, *G* = group), and cohesiveness during foraging (*G* = gregarious, *D* = dispersed) as well as suggested function of latrine use (*1* = territory demarcation, *2* = resource defense, *3* = centers of information exchange, *4* = reproductive signaling, *5* = mate defense/intrasexual competition, *6* = signaling of social status)

Order	Species	Common name	Habitat	Activity	Social organization	Cohesion	Territoriality	Function	Reference
Artiodactyla	*Alcelaphus buselaphus*	Hartebeest	T	D	G	G	Yes		Gosling (1974)
	*Cervus eldi*	Eld’s deer	T	N/C	G	G	No		Wemmer and Montali (1988)
	*Damaliscus korrigum*	Topi	T	N/D	G	G	Yes	1	Gosling (1987)
	*Gazella dorcas*	Dorcas gazelle	T	N/D/C	P/G	G	Yes		Essghaier and Johnson (1981)
	*Gazella gazella*	Mountain gazelle	T	D	G	G	Yes		Wronski and Plath (2010)
	*Gazella granti*	Grant’s gazelle	T	N/D	G	G	Yes	1	Estes (1991)
	*Gazella thomsoni*	Thomson’s gazelle	T	N/D	G	G	Yes		Walther (1978)
	*Hydropotes inermis*	Water deer	T	C	S	D	Yes		Sun et al. (1994)
	*Lama guanicoe*	Guanaco	T	D	G	G	Yes		Henriquez (2004)
	*Madoqua guentheri*	Guenther’s dik-dik	T	N/D	P	G	Yes	1	Ono et al. (1988)
	*Madoqua kirkii*	Kirk’s dik-dik	T	N/D	P	G	Yes	3	Hendrichs and Hendrichs (1971)
	*Mazama americana*	Red brocket deer	T	N/D	S/P	D	Yes		Rivero et al. (2004)
	*Mazama gouazoubira*	Brown brocket deer	T	N	S	D	Yes	3,5	Black-Decima and Santana (2011)
	*Moschus chrysogaster*	Alpine musk deer	T	N	G	D	Yes		Qureshi et al. (2004)
	*Moschus moschiferus*	Siberian musk deer	T	N	G	D	Yes		Green (1987)
	*Muntiacus muntjak*	Indian muntjac	T	N/D	S	D	Yes	1	Dubost (1971)
	*Muntiacus reevesi*	Chinese muntjac	T	N/D	S	D	Yes	1	Dubost (1970)
	*Oreotragus oreotragus*	Klipspringer	T	D	P	G	Yes	1	Roberts and Lowen (1997)
	*Ourebia ourebi*	Oribi	T	D	S/P/G	G	Yes	1,3	Brashares and Arcese (1999)
	*Pudu puda*	Southern pudu	T	N/D	S	D	Yes		MacNamara and Eldridge (1987)
	*Raphicerus campestris*	Steinbuck	T	D	P	D	Yes	3	Kingdon (1982)
	*Tragelaphus scriptus*	Bushbuck	T	N/C	G	D	Yes	3,4	Wronski et al. (2006)
	*Vicugna pacos*	Alpaca	T	D	G	G	Yes		McGregor and Brown (2010)
Perissodactyla	*Ceratotherium simum*	White rhinoceros	T	N/D	S/G	G	Yes		Owen-Smith (1975)
	*Diceros bicornis*	Black rhinoceros	T	N/D	S	D	Yes		Linklater et al. (2013)
	*Rhinocerus unicornis*	Indian rhinoceros	T	N/D	S	D	Yes		Dinerstein and Wemmer (1988)
	*Tapirus terrestris*	South American tapir	T	N/C	S	D	Yes		Fragoso et al. (2003)
Carnivora	*Bassariscus astutus*	Ring-tailed cat	T	N/C	S	D	Yes		Barja and List (2006)
	*Canis aureus*	Golden jackal	T	N/D	G	D	Yes		Macdonald (1980)
	*Canis latrans*	Coyote	T	N/D	S/P/G	D,G	Yes		Ralls and Smith (2004)
	*Canis simensis*	Ethopian wolf	T	D	G	D	Yes		Sillero-Zubiri and Macdonald (1998)
	*Civettictis civetta*	African civet	T	N	S	D	Yes		Bearder and Randall (1978)
	*Crocuta crocuta*	Spotted hyena	T	N	G	G	Yes		Gorman and Mills (1984)
	*Genetta genetta*	Common genet	T, A	N	S/P	D	Yes	4,5	Barrientos (2006)
	*Hyaena brunnea*	Brown hyena	T	N	G	G	Yes	1	Mills et al. (1980)
	*Hyaena hyaena*	Striped hyena	T	N	G	D	Yes		Macdonald (1980)
	*Lontra canadensis*	River otter	T, AQ	N/C	G	G	Yes	6	Rostain et al. (2004)
	*Martes martes*	Pine marten	T,A	N	S	D	Yes		Barja et al. (2011)
	*Meles meles*	European badger	T	N/C	G	D	Yes	1,2,4,5	Roper et al. (1993), Balestrieri et al. (2011)
	*Nyctereutes procyonoides*	Raccoon dog	T	N	P	D	No	3	Ikeda (1984)
	*Procyon lotor*	Northern raccoon	T	N	G	D	Variable		Brown and Macdonald (1985)
	*Proteles cristatus*	Aardwolf	T	N	P	D	Yes		Nel and Bothma (2002)
	*Pteronura brasiliensis*	Giant otters	T, AQ	D	G	G	Yes		Leuchtenberger and Mourão (2009)
	*Suricata suricatta*	Meerkats	T	D	G	G	Yes	1,5	Jordan et al. (2007)
	*Urocyon cinereoargenteus*	Gray fox	T	N/C	P	D	Yes		Trapp (1978)
	*Vulpes macrotis*	Kit fox	T	N	P	D	Yes		Ralls and Smith (2004)
	*Vulpes velox*	Swift fox	T	N	P	D	Yes	1	Darden et al. (2008)
Dasyuromorphia	*Dasyurus geoffroii*	Western quoll	T	N/C	S	D	Yes		Serena and Soderquist (1989)
	*Dasyurus hallucatus*	Northern quoll	T	N	S	D	No		Oakwood (2002)
	*Dasyurus maculatus*	Tiger quoll	T	N	S	D	Yes		Ruibal et al. (2010)
	*Myrmecobius fasciatus*	Numbat	T	D	S	D	Yes	1	Hogan et al. (2013)
	*Sarcophilus harrisii*	Tasmanian devil	T	N	S	D	No		Pemberton (1990)
Diprotodontia	*Petropseudes dahli*	Rock-haunting possum	T	N	P	G	Yes		Runcie (2004)
Hyracoidea	*Dendrohyrax arboreus*	Southern tree hyrax	A	N/D	S/P	D	Yes		Milner and Harris (1999)
	*Dendrohyrax validus*	Eastern tree hyrax	A	N	?	D	Yes		Topp-Jørgensen et al. (2008)
	*Heterohyrax brucei*	Yellow-spotted rock hyrax	T	D	G	G	Yes		Barry and Shoshani (2000)
	*Procavia capensis*	Rock hyrax	T	D	G	G	Yes		Meadows et al. (2010)
Lagomorpha	*Oryctolagus cuniculus*	European rabbit	T	N	G	G	Yes		Sneddon (1991)
Primates	*Alouatta caraya*	Black howler monkey	A	D	G	G	Yes		Bravo and Zunino (2000)
	*Alouatta seniculus*	Red howler monkey	A	D	G	G	Yes		Julliot (1996)
	*Ateles geoffroyi*	Geoffroy’s spider monkey	A	D	G	G	Yes		González-Zamora et al. (2012)
	*Cheirogaleus major*	Greater dwarf lemur	A	N	P	D	Yes		Petter (1962)
	*Cheirogaleus medius*	Fat-tailed dwarf lemur	A	N	P	D	Yes		Petter (1962)
	*Hapalemur griseus*	Lesser bamboo lemur	A	D	G	G	Yes	2,4,5	Irwin et al. (2004)
	*Hapalemur meridionalis*	Southern lesser bamboo lemur	A	D	G	G	Yes	1,2	Eppley and Donati (2010)
	*Lagothrix lagotricha*	Woolly monkey	A	D	G	G	Yes		Yumoto et al. (1999)
	*Lepilemur leucopus*	White-footed sportive lemur	A	N	P	D	Yes	3,5	This study
	*Lepilemur wrightae*	Wright’s sportive lemur	A	N	P	D	Yes	2,4,5	Irwin et al. (2004)
Rodentia	*Arvicola terrestris*	Water vole	T, AQ	N	S	D	Yes	4	Woodroffe and Lawton (1990)

## Electronic supplementary material

Below is the link to the electronic supplementary material.

ESM 1 (DOCX 21 kb)
