# Supplementary material for: Maintenance of familiarity and social bonding via communal latrine use in a solitary primate (Lepilemur leucopus)
Source: Behav Ecol Sociobiol. 2014 Oct 16;68(12):2043–58. doi: 10.1007/s00265-014-1810-z (PMC4220112; doi:10.1007/s00265-014-1810-z)
Supplement: Supplementary file 1 — (DOCX 21 kb) [file 265_2014_1810_MOESM1_ESM.docx]

**Appendix A** Summary of continuous focal animal observations (hours = time in sight) conducted throughout the year on the focal animals belonging to seven social units of *Lepilemur leucopus* (Season: 1 = early wet, 2 = late wet, 3 = early dry, 4 = late dry).

| **Social Unit** | Date | Season | **female** | hours | **male** | hours |
| --- | --- | --- | --- | --- | --- | --- |
| **1** | 05.11.2011 | 1 | **f1B** | 10:13:26 |  |  |
| **1** | 08.01.2012 | 1 | **f1B** | 9:22:17 |  |  |
| **1** | 21.02.2012 | 2 | **f1B** | 9:41:13 |  |  |
| **1** | 21.04.2012 | 2 | **f1B** | 11:08:29 |  |  |
| **1** | 05.06.2012 | 3 | **f1B** | 11:31:46 | **m10** | 11:00:02 |
| **1** | 11.07.2012 | 3 | **f1B** | 10:36:17 | **m10** | 10:58:23 |
| **1** | 26.08.2012 | 4 | **f1B** | 9:52:43 | **m10** | 10:48:33 |
| **1** | 04.10.2012 | 4 | **f1B** | 9:57:08 | **m10** | 8:52:39 |
| **2** | 23.10.2011 | 1 | **f2** | 10:23:35 |  |  |
| **2** | 05.12.2011 | 1 | **f2** | 9:35:30 | **m9** | 10:05:03 |
| **2** | 06.02.2012 | 2 | **f2** | 8:43:11 | **m9** | 9:59:52 |
| **2** | 23.03.2012 | 2 | **f2** | 10:51:56 | **m9** | 10:01:42 |
| **2** | 09.05.2012 | 3 | **f2** | 10:15:40 | **m9** | 11:09:34 |
| **2** | 27.06.2012 | 3 | **f2** | 11:12:43 | **m9** | 11:30:45 |
| **2** | 17.08.2012 | 4 | **f2** | 10:52:33 | **m9** | 11:08:09 |
| **2** | 26.09.2012 | 4 | **f2** | 10:39:33 | **m9** | 10:32:36 |
| **3** | 14.11.2011 | 1 |  |  | **m3** | 8:34:09 |
| **3** | 23.12.2011 | 1 | **f3** | 8:55:11 | **m3** | 9:27:30 |
| **3** | 01.11.2011 | 1 | **f3** | 10:22:56 |  |  |
| **3** | 02.02.2012 | 1 |  |  | **m3** | 7:34:06 |
| **3** | 13.03.2012 | 2 | **f3** | 9:55:23 |  |  |
| **3** | 31.03.2012 | 2 | **f3** | 9:40:59 | **m3** | 9:35:58 |
| **3** | 04.05.2012 | 3 | **f3** | 10:54:01 | **m3** | 10:12:30 |
| **3** | 23.06.2012 | 3 | **f3** | 9:58:28 | **m3** | 11:24:59 |
| **3** | 01.08.2012 | 4 | **f3** | 11:04:32 | **m3** | 10:57:07 |
| **3** | 12.09.2012 | 4 | **f3** | 10:08:31 | **m3** | 11:03:17 |
| **4** | 13.12.2011 | 1 | **f4** | 8:53:26 | **m4** | 8:23:19 |
| **4** | 25.01.2012 | 1 | **f4** | 9:20:19 | **m4** | 9:19:43 |
| **4** | 19.03.2012 | 2 | **f4** | 10:39:27 | **m4** | 10:02:49 |
| **4** | 30.04.2012 | 2 | **f4** | 10:19:40 | **m4** | 11:24:32 |
| **4** | 18.06.2012 | 3 | **f4** | 11:38:01 | **m4** | 12:00:43 |
| **4** | 25.07.2012 | 3 | **f4** | 9:55:08 | **m4** | 11:27:02 |
| **4** | 08.09.2012 | 4 | **f4** | 10:41:58 | **m4** | 10:43:59 |
| **4** | 18.10.2012 | 4 | **f4** | 10:11:10 | **m4** | 10:26:48 |
| **5** | 23.11.2011 | 1 | **f5** | 9:19:56 | **m5** | 9:22:27 |
| **5** | 03.01.2012 | 1 | **f5** | 8:43:59 | **m5** | 8:34:58 |
| **5** | 25.02.2012 | 2 | **f5** | 9:25:49 | **m5** | 7:11:39 |
| **5** | 09.04.2012 | 2 | **f5** | 10:16:38 | **m5** | 10:41:26 |
| **5** | 10.06.2012 | 3 | **f5** | 11:26:29 | **m5** | 11:43:06 |
| **5** | 15.07.2012 | 3 | **f5** | 11:28:27 | **m5** | 11:19:01 |
| **5** | 30.08.2012 | 4 | **f5** | 10:49:42 | **m5** | 10:38:59 |
| **5** | 08.10.2012 | 4 | **f5** | 10:38:38 | **m5** | 10:22:12 |
| **6** | 27.10.2011 | 1 | **f6** | 10:30:56 |  |  |
| **6** | 09.12.2011 | 1 | **f6** | 9:34:32 | **m6** | 9:21:22 |
| **6** | 28.12.2011 | 1 |  |  | **m6** | 8:24:32 |
| **6** | 10.02.2012 | 2 | **f6** | 8:54:54 | **m6** | 7:57:50 |
| **6** | 27.03.2012 | 2 | **f6** | 10:36:20 | **m6** | 10:41:52 |
| **6** | 30.05.2012 | 3 | **f6** | 11:29:25 | **m6** | 9:56:14 |
| **6** | 02.07.2012 | 3 | **f6** | 11:13:23 | **m6** | 11:19:13 |
| **6** | 21.08.2012 | 4 | **f6** | 11:08:09 | **m6** | 10:46:40 |
| **6** | 30.09.2012 | 4 | **f6** | 11:46:49 | **m6** | 9:59:26 |
| **7** | 18.11.2011 | 1 | **f7** | 9:12:27 | **m7** | 9:28:42 |
| **7** | 21.01.2012 | 1 | **f7** | 9:12:05 | **m7** | 7:59:54 |
| **7** | 04.03.2012 | 2 | **f7** | 9:54:23 | **m7** | 10:23:34 |
| **7** | 25.04.2012 | 2 | **f7** | 11:06:15 | **m7** | 11:05:35 |
| **7** | 14.06.2012 | 3 | **f7** | 12:01:14 | **m7** | 11:57:52 |
| **7** | 20.07.2012 | 3 | **f7** | 10:49:22 | **m7** | 12:12:28 |
| **7** | 03.09.2012 | 4 | **f7** | 10:34:57 | **m7** | 11:01:25 |
| **7** | 12.10.2012 | 4 | **f7** | 10:28:29 | **m7** | 10:20:33 |

article title: **Maintenance of familiarity and social bonding via communal latrine use in a solitary primate (*Lepilemur leucopus*)**

journal name: **Behavioral Ecology and Sociobiology**

author names: **Iris Dröscher^1^, Peter M. Kappeler^1,2^**

affiliation:

**^1^ Behavioral Ecology & Sociobiology Unit, German Primate Center, Kellnerweg 4, 37077 Göttingen, Germany**

**^2^ Department of Sociobiology/Anthropology, Johann-Friedrich-Blumenbach Institute of Zoology & Anthropology, University of Göttingen, Kellnerweg 6, 37077 Göttingen, Germany**

e-mail address of the corresponding author: **iris.droescher@gmail.com**
